# Prokaryotic and eukaryotic microbial diversity from three soda lakes in the East African Rift Valley determined by amplicon sequencing

**DOI:** 10.3389/fmicb.2022.999876

**Published:** 2022-12-08

**Authors:** Oliyad Jeilu, Amare Gessesse, Addis Simachew, Eva Johansson, Erik Alexandersson

**Affiliations:** ^1^Institute of Biotechnology, Addis Ababa University, Addis Ababa, Ethiopia; ^2^Department of Plant Breeding, Swedish University of Agricultural Sciences, Lomma, Sweden; ^3^Department of Biological Sciences and Biotechnology, Botswana International University of Science and Technology, Palapye, Botswana; ^4^Department of Plant Protection Biology, Swedish University of Agricultural Sciences, Lomma, Sweden

**Keywords:** microbial diversity, extremophiles, haloalkalophiles, operational taxonomic units, soda lakes

## Abstract

Soda lakes are unique poly-extreme environments with high alkalinity and salinity that support diverse microbial communities despite their extreme nature. In this study, prokaryotic and eukaryotic microbial diversity in samples of the three soda lakes, Lake Abijata, Lake Chitu and Lake Shala in the East African Rift Valley, were determined using amplicon sequencing. Culture-independent analysis showed higher diversity of prokaryotic and eukaryotic microbial communities in all three soda lakes than previously reported. A total of 3,603 prokaryotic and 898 eukaryotic operational taxonomic units (OTUs) were found through culture-independent amplicon sequencing, whereas only 134 bacterial OTUs, which correspond to 3%, were obtained by enrichment cultures. This shows that only a fraction of the microorganisms from these habitats can be cultured under laboratory conditions. Of the three soda lakes, samples from Lake Chitu showed the highest prokaryotic diversity, while samples from Lake Shala showed the lowest diversity. *Pseudomonadota* (*Halomonas*), *Bacillota* (*Bacillus*, *Clostridia*), *Bacteroidota* (*Bacteroides*), *Euryarchaeota* (*Thermoplasmata*, *Thermococci*, *Methanomicrobia*, *Halobacter*), and *Nanoarchaeota* (*Woesearchaeia*) were the most common prokaryotic microbes in the three soda lakes. A high diversity of eukaryotic organisms were identified, primarily represented by *Ascomycota* and *Basidiomycota*. Compared to the other two lakes, a higher number of eukaryotic OTUs were found in Lake Abijata. The present study showed that these unique habitats harbour diverse microbial genetic resources with possible use in biotechnological applications, which should be further investigated by functional metagenomics.

## Introduction

Extremophiles are organisms that are able to thrive in environments considered extreme, at least from the human perspective. These environments include extreme physical factors such as pressure, radiation, and temperature and geochemical factors such as desiccation, pH, and salinity (Shrestha et al., 2018; Zhu et al., 2020). While most extremophiles are able to exist and grow under a single extreme environmental condition, a few can grow under multiple extreme conditions. Soda lakes, with both high salinity and alkalinity, are good examples of ecosystems with double extremes. Though such polyextreme environments were expected to have limited biodiversity, recent studies showed that soda lakes have huge microbial biodiversity (Sorokin et al., 2014; Ersoy Omeroglu et al., 2021).

Soda lakes have worldwide distribution (Andreote et al., 2018), with many such ecosystems found along the East African Rift Valley (Schagerl, 2016). Investigations on the microbial diversity of Ethiopian soda lakes have resulted in the identification of diverse groups of (halo)alkaliphilic bacterial and archaeal phyla (Martins et al., 2001; Lanzén et al., 2013; Simachew et al., 2016; Islam et al., 2021). Most studies of extremophiles originating from the Ethiopian soda lakes have so far been based on culture-dependent systems with the aim to selectively isolating pure culture strains to understand their physiology or to evaluate their potential in biotechnological applications (Gessesse and Gashe, 1997; Martins et al., 2001; Haile and Gessesse, 2012). A major drawback of this approach is that only a small fraction of the microorganisms are able to grow in standard culture media (Bodor et al., 2020). This makes a culture-dependent approach, in addition to being laborious and time-consuming (Maukonen et al., 2003; Kambura et al., 2016; Rosenthal et al., 2017), not reliable for studying microbial diversity. Therefore, a better and more reliable estimate of microbial diversity in any natural habitat can be obtained using culture-independent methods using molecular techniques.

Although the microbial diversity from the three Ethiopian soda lakes was recently studied using culture-independent methods, these were based on the analysis of clone libraries (Tafesse, 2014) or sequencing platforms that produce low read depths (Lanzén et al., 2013; Simachew et al., 2016). Due to the low sequence capture in the cloning step, while creating DNA libraries, and the low number of amplicon sequence reads generated, these techniques have inherent limitations in accurately reflecting the true picture of microbial diversity (Burke and Darling, 2016; Besser et al., 2018). Moreover, in previous culture-independent studies, little attention was given to studying the microbial diversity of eukaryotes from soda lakes (e.g., Lanzén et al., 2013). On the other hand, studies of other extreme environments have demonstrated the presence of extremophilic fungi that grow and reproduce at elevated temperatures or under alkaline and high salinity (Orwa et al., 2020). Such extremophilic eukaryotes, apart from their ecological function (de Oliveira and Rodrigues, 2019), may serve as potential sources of new biotechnological products (Ali et al., 2019).

The Ethiopian soda lakes are known for their high primary productivity, which in fact, represents one of the highest for any natural environment (Wood et al., 1984). Studies based on culture-independent and high-throughput next-generation sequencing have the potential to increase our understanding of the diversity of the microbiota in these ecosystems and to further elucidate the possible uses in biotechnological applications. The main aim of this study was to estimate the diversity of prokaryotic and eukaryotic microorganisms in samples of three soda lakes of the East African Rift Valley by a culture-independent analysis utilizing Illumina HiSeq sequencing. In addition, we sequenced the microbial communities after the samples were cultured in the laboratory to explore whether some of these organisms could be maintained in controlled conditions.

## Materials and methods

### Sampling sites, sample collection and preparation

**Figure 1:**
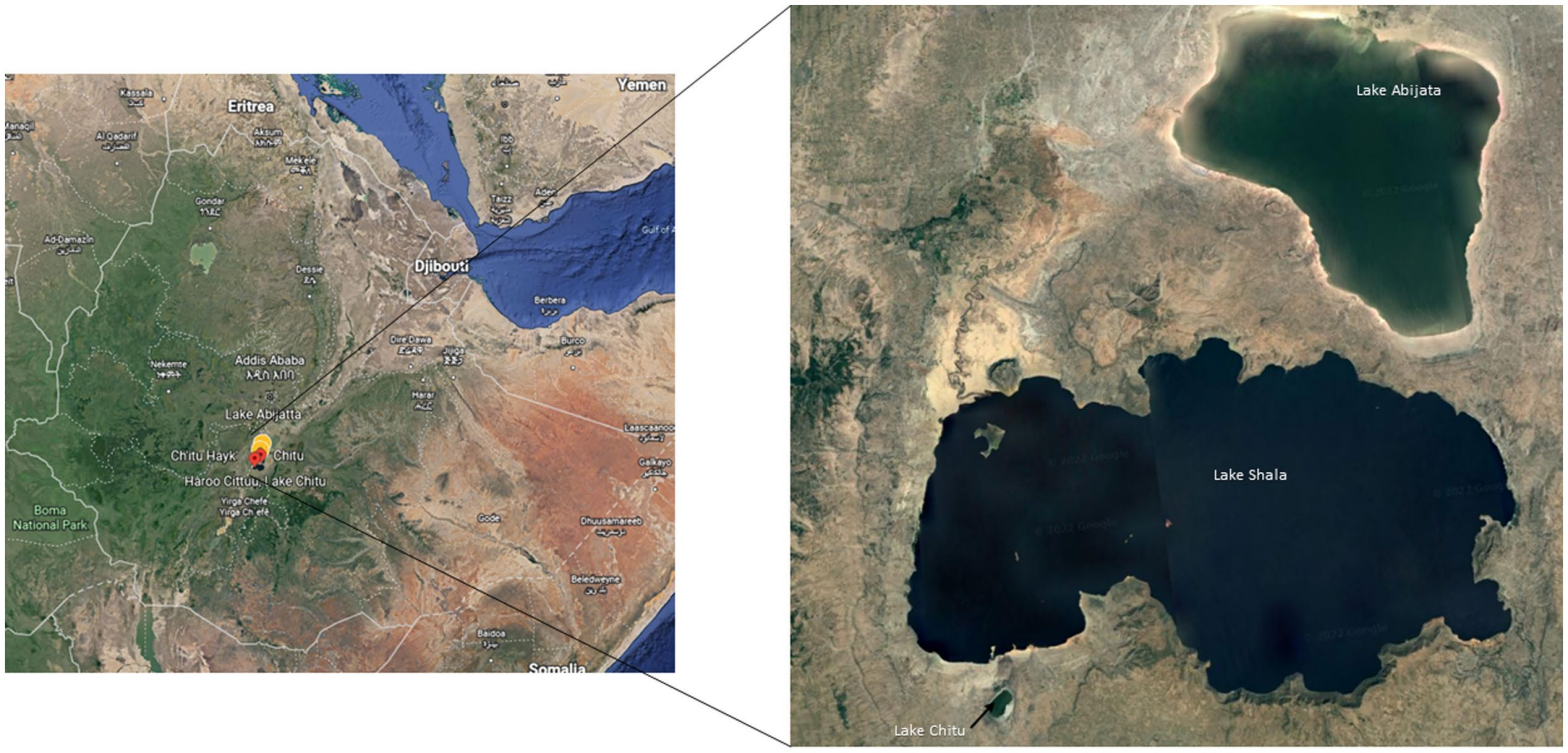
Sampling locations of the three soda lakes in the Ethiopian Rift Valley (image adapted from Google map 7^th^ September 2021).

Water and sediment samples from three soda lakes in the East African Rift Valley, Lakes Abijata, Chitu, and Shala, were collected using sterile Niskin bottles (Ocean Scientific International Ltd.) and polyethylene bags, respectively (Figure 1). Samples from 19 sites were collected in triplicates (Supplementary Table 1). The sampling sites were selected randomly based on the accessibility of the lakes. Geographical coordinates, depths, pH, and salinity were measured using GPS, Ekman grab attached with meter, pH meter (OAKTON-pH110), and refractometer (HHTEC; Supplementary Table 1). The water samples were divided into two parts, with one part filtered within 24 h after sample collection, using a polycarbonate filter membrane (22 μm pore size, 47 mm diameter; GE) to collect the microbes for DNA extraction.

For the enrichment culture study, the second part of the water samples was again divided into two halves, with one half filtered as mentioned above and the other half left unfiltered. The filtered lake water (brine) samples were used as a culture media and with unfiltered water samples as inoculum (5% v/v). The sediment samples were suspended in distilled water first, and once the sediment particles settled, the water was used as an inoculum. The inoculated brine was then incubated on a shaker (INFORS HT Ecotron Incubator; 120 rpm) for up to 7 days at room temperature. Until the seventh day, aliquots of each sample were taken every 24 h. The aliquots were centrifuged for 12 min at 4500 × g, and the cell pellets were pooled and saved for DNA extraction.

### DNA extraction

DNA was extracted from the water and enrichment culture samples according to Øvreås et al. (2003) with some modifications as described below. Briefly, 250 μl of Lysozyme (1 mg/ml)/RNase solution (0.5 mg/ml, Thermo Scientific) was added to the microbial biomass on polycarbonate filter membrane followed by incubation for 15 min at 37°C. After that, 10 μl of Proteinase K (1 mg/ml, Thermo Scientific) was added to the filter, which again was incubated for 15 min at 37°C, followed by the addition of 250 μl pre-heated sodium dodecyl sulfate (SDS) solution (10% w/v) before further incubation for 15 min at 55°C. Finally, 80 μl of NaCl (5 M) and 100 μl of cetyltrimethylammonium bromide (CTAB; 1%) were added and incubated for 10 min at 65°C followed by the addition of 750 μl chloroform/isoamyl alcohol (24:1). The mixture was centrifuged at 12,000 × g for 15 min, and the aqueous layer was transferred into Eppendorf tubes and precipitated with 0.6 volume of isopropanol. After centrifugation at 12,000 × g for 15 min, the pellet was washed with 70% ethanol, dried at room temperature, and dissolved in TE buffer (pH 8.0).

DNA from the sediment samples was extracted according to Verma and Satyanarayana (2011) with some modifications as described below. About 10 g of sediment placed in sterile Falcon tubes were suspended in 13.5 ml of extraction buffer [1% CTAB, (w/v), 100 mM Tris (pH 8.0), 100 mM NaH_2_PO_4_ (pH 8.0), 100 mM EDTA and 1.5 M NaCl]. Thereafter, 50 μl of proteinase K (10 mg/ml, Thermo Scientific) was added, followed by incubation for 30 min at 37°C. After that, 1.5 ml of 20% SDS was added, followed by incubation at 65°C for 2 h, with gentle inversion every 15 min. The samples were then centrifuged at 4,000 × g for 20 min at room temperature, to separate the sediment residues from the cell lysates, which were transferred to new sterile centrifuge tubes. An equal volume of phenol/chloroform/isoamyl alcohol (25:24:1) was added to the cell lysates, and samples were centrifuged at 16,000 × g for 5 min. The aqueous supernatant was again transferred to sterile centrifuge tubes, and an equal volume of chloroform was added to the tubes. After mixing, the tubes were centrifuged at 16,000 × g for 5 min. The aqueous layer was transferred to new sterile tubes, and the DNA was precipitated by adding about 0.6 volumes of isopropanol and recovered by centrifuging at 16,000 × g for 10 min. The pellet was washed with 70% ethanol and centrifuged at 16,000 × g for 5 min. Finally, the pellet was air-dried and dissolved in TE buffer (pH 8.0).

### DNA purification and pooling

The extracted DNA was further purified using the DNeasy PowerSoil DNA extraction kit (QIAGEN), according to the manufacturer’s instructions. About 250 μl aluminium chloride solution was added to each 50 μl DNA sample, which were then incubated for 5 min at room temperature. Thereafter, the samples were centrifuged at 15,000 × g for 2 min, and the supernatant was mixed with binding solution and loaded into the spin column, which was centrifuged at 10,000 × g for 1 min to discard the flow-through. The bound DNA was washed with 70% ethanol a few times and eluted with 50 μl of TE buffer (pH 8.0). Quantity and quality of DNA purified were checked using NanoDrop (260/280 ratio > 1.7; Thermo Scientific) and agarose gel electrophoresis (Thermo Scientific). The DNA of each biological triplicate were pooled before amplification of 16S rRNA and ITS genes.

### Amplification of 16S rRNA and ITS gene sequences and sequencing

The V4 region of the 16S rRNA gene of the prokaryotic community was amplified using the primer sets 515F (5’-GTGCCAGCMGCCGCGGTAA-3′) and 806R (5’-GGACTACHVGGGTWTCTAAT-3′; Walters et al., 2016). In addition, ITS primer sets ITS1F (5’-CTTGGTCATTTAGAGGAAGTAA-3′) and ITS2R (5′- GCTGCGTTCTTCATCGATGC-3′) were used to amplify the ITS region of the fungal community (Op De Beeck et al., 2014). The 16S rRNA and ITS amplicon products were sequenced at BGI in Hong Kong using Illumina HiSeq Sequencing with a 2 × 50 pair end approach.

### 16S rRNA and ITS sequence quality control and filtering

The 16S rRNA and ITS sequences were analyzed using the Nextflow computational pipeline ampliseq v1.1.2.^1^ Briefly, raw sequencing reads were quality checked using FastQC (Andrews, 2010), followed by trimming adaptor sequences from the reads using cutadapt v2.7 (Martin, 2011). Quality distribution of trimmed reads was then analyzed using tools available in the QIIME2 software package v2019.10 (Bolyen et al., 2019). After quality filtering, the sequences were denoised, dereplicated, and filtered for chimeric sequences using pair-ended DADA2 (Callahan et al., 2016), resulting in exact amplicon sequence variants (ASVs) tables.

### OTU clustering and taxonomic classification

Amplicon sequence variants (ASVs) identified in 16S rRNA gene sequences were taxonomically classified from phylum to species level, and clustered with 99% similarity using the SILVA v132 database (Quast et al., 2013). Following the taxonomic classification of ASVs to OTUs (Operational Taxonomic Units), the OTUs classified as Mitochondria or Chloroplast were removed. The taxonomic classification of ASVs inferred in ITS sequences was performed using UNITE v8.99 database of ITS sequences, with the same parameters used for 16S rRNA gene sequences (Nilsson et al., 2019).

### Diversity analysis and visualization

Analyses and visualizations of amplicon sequence variants (ASVs) were carried out *via* R (v4.0.4; R Core Team, 2014). The sequence reads were normalized to a minimal library size using the rarefying method (Weiss et al., 2017). The analysis of diversity measures such as alpha diversity, beta diversity, and ordination and visualization were performed using the R packages phyloseq v1.34.0 (McMurdie and Holmes, 2013), microbiome v1.12.0 (Lahti et al., 2017), and vegan v2.5.7 (Dixon, 2003).

## Results

### Physico-chemical properties of the soda lakes

Salinity and pH differed between the lakes at the time of sampling, with the lowest salinity level of 3% in Lake Shala and the highest level of 15% in Lake Abijata, while the lowest pH of 9.3 was found in Lake Shala and the highest pH of 10.0 in Lake Chitu (Table 1; Supplementary Table 1).

**Table 1:** General features of the soda lakes, the detected prokaryotic OTUs and sequence reads of the environmental samples.

Lake	pH	Salinity (%)	OTUs detected			Number of reads			Unique OTUs*	Shared OTUs	% OTU Occurrence***	Domain		
			Water	Sediment	Total	Water	Sediment	Total	(%)	(%)		Bacteria	Archaea	Unidentified
Abijata	9.5	15	459	1,168	1,276	251,101	353,866	604,967	18.2	17.2	35.4	1,166	110	0

Chitu	10	6	2,659	355	2,692	721,627	266,427	988,054	55.1	19.7	74.7	2,285	406	1
Shala	9.3	3	280	359	512	215,467	50,742	266,209	6.4	7.8	14.2	503	9	0
Total			3,603		3,603	1,859,230		1,859,230	79.7	20.3	100	3,603		

*Unique OTUs refers to those that are detected only in samples of one lake.

**Shared OTU refers to those that occur in samples of two or all three lakes.

***OTU occurrence refers to the proportion of OTUs detected in samples of each lake out of the total OTU detected.

### Prokaryotic microbial sequence reads and OTUs

About 1,859,230 prokaryotic sequence reads (between 40,413 and 397,096 per sample) were captured in samples from the three investigated lakes using 16S rRNA sequencing. From these sequence reads, a total of 3,603 prokaryotic OTUs were identified (Table 1).

About 80% (2,872) of the OTUs were unique to samples of one lake, while the remaining 20% (731 OTUs) were detected in samples from two or all three lakes (Table 1). Samples of Lake Chitu showed the highest OTU abundance among the lakes, accounting for 75% of all the OTUs detected in the samples of all the three lakes, while samples of Lake Shala showed the lowest OTU abundance (14%) with only 512 OTUs (Table 1).

The total sequence reads of water and sediment samples ranged from 266,209 to 988,054 in the three lakes (Table 1). For Lake Abijata, only 36% of the OTUs were detected in the water samples, while the highest proportion of OTUs (92%) were detected from the sediment samples. In contrast, 99% of the OTUs from, Lake Chitu were detected in the water samples and only 13% were detected in the sediment samples (Table 1). For Lake Shala, the division of OTUs between water and sediment samples were more equal, with 55% of the OTUs detected in the water samples and 70% of the OTUs from sediment.

**Figure 2:**
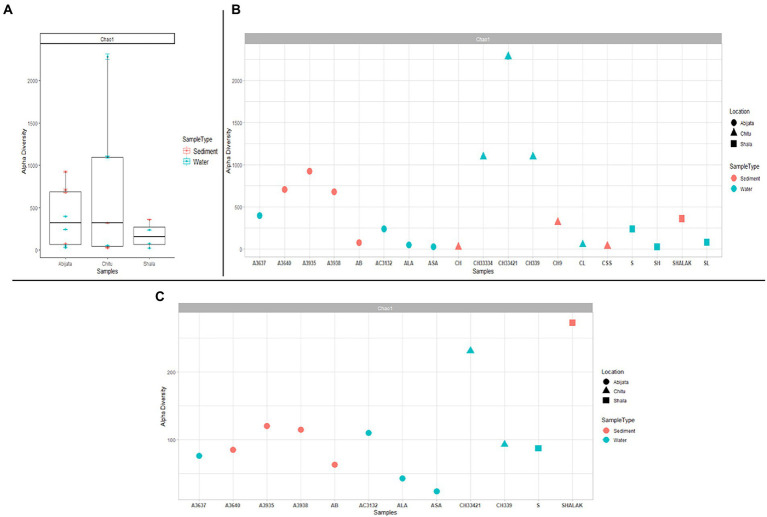
Alpha diversity for prokaryotes in the lakes **(A)** either comparing between the three lakes **(B)** or as a diversity between all samples. **(C)** Alpha diversity measures between samples for eukaryotes. The sample names are on the x-axis and the estimated diversity on the y-axis.

Samples of Lake Chitu showed the highest alpha diversity among the lakes, while samples of Lake Shala had the lowest diversity (Figure 2A). Species diversity differed, with the highest species diversity in the Lake Chitu samples, CH33334, CH33421, and CH339, which were all water samples, although high species diversity was also noted for sediment samples from Lake Abijata (Figure 2B).

### Shared and unique prokaryotic OTUs within and between the soda lakes

**Figure 3:**
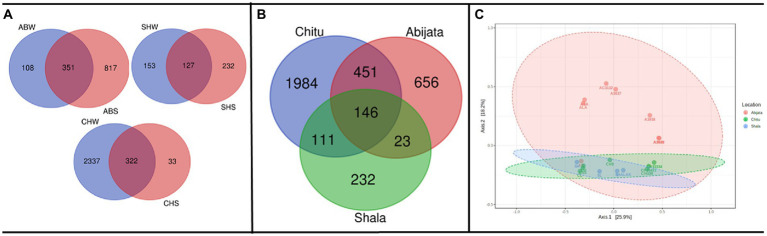
Shared and unique prokaryotic OTUs **(A)** between sample types in the three soda lakes; ABW: Abijata water samples, ABS: Abijata Sediment samples, CHW: Chitu water samples, CHS: Chitu sediment samples, SHW: Shala water samples, SHS: Shala sediment samples **(B)** between the lakes **(C)** PCoA using Bray distance; Statistical significance was found out using Analysis of group Similarities [ANOSIM] R: 0.32947; value of *p* <0.005. Sample names (Water samples: A3637, AB, AC3132, ALA, ASA, CH33334, CH33421, CH339, CL, S, SH, SL; Sediment samples: A3640, A3935, A3938, CH, CH9, CSS, SHALAK).

As presented in Figure 3A, a relatively low proportion (28, 12 and 25%, respectively) of the OTUs were shared between water (W) and sediment (S) samples in the Abijata (AB), Chitu (CH) and Shala (SH) lakes. Similarly, a rather low proportion of the OTUs were shared among the different lakes (Figure 3B), e.g., only 146 OTUs (about 4% of the total) were shared between samples of the three lakes, and only a total of 451 OTUs (about 12%) were shared between samples of Lake Abijata and Lake Chitu. As pointed out above, samples of Lake Chitu had the highest number of OTUs among samples of the lakes, but also, the proportion of unique OTUs (74%) was the highest (Figure 3B). Analysis by one-way ANOVA showed that the OTUs between the lakes were significantly different (*F*-value = 10.1 *p* < 0.05). A Principal Coordinates Analysis (PCoA) displayed a spread of the samples’ microbial composition from the different lakes along the first principal coordinate (explaining 25.9% of the variation), visualizing the variation in OTUs between samples also within each of the three lakes. The second principal coordinate divided the Lake Shala and Chitu samples from the Lake Abijata samples, indicating a higher similarity among Lake Shala and Lake Chitu OTUs than for Lake Abijata OTUs, although the second coordinate had a relatively low level of explanation of variation (18.2%; Figure 3C).

### Prokaryotic taxonomic distribution

With 99% sequence similarity, 87% of OTUs (3,120) were identified as bacteria, 13% (482) as archaea, while a single OTU was not classified as either Bacteria or Archaea using the SILVA database (Table 1).

**Figure 4:**
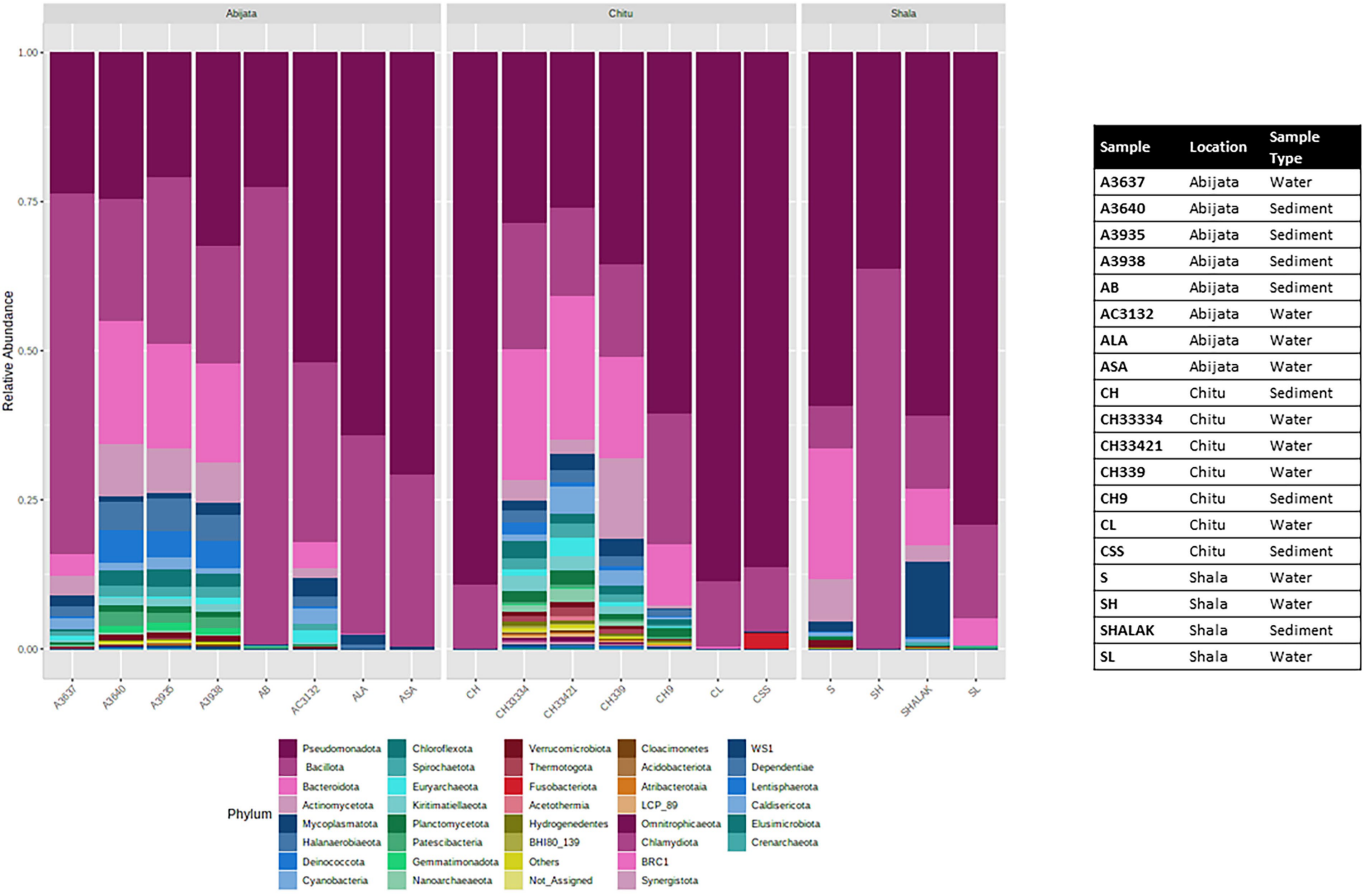
Stacked plot showing the taxonomic composition of the community at the phylum level (small taxa with read counts <100 merged as others).

The OTUs were further classified into 58 phyla (50 identified bacterial phyla, 7 identified archaeal phyla and 1 unidentified phylum; Figure 4), where three phyla, *Pseudomonadota, Bacillota,* and *Bacteroidota,* were the most abundant and represented 49% of the OTUs (Figure 4) whereas *GN01, Rokubacteria, Asgardaeota, Fibrobacterota, MAT-CR-M4-B07, FCPU426, Chrysiogenota*, PAUC34f, *WS2,* and *Modulibacteria* had the lowest abundance (Supplementary Table 2).

### Bacterial community composition

For Lake Abijata and Lake Shala, the phylum *Pseudomonadota* was the most dominant in both water and sediment samples, followed by *Bacillota*, while for Lake Chitu, the phylum *Bacillota* was represented by slightly more OTUs in both water and sediment samples than *Pseudomonadota. Bacteroidota* was the third most abundant phylum in samples in all of the lakes (Table 2; Figure 4; Supplementary Table 2).

**Table 2:** Distribution of bacterial phylum of the environmental samples identified through cuture-independent investigation.

**Phylum**	**All**		**Abijata**		**Chitu**		**Shala**	
	OTUs	RA%	OTUs	RA%	OTUs	RA %	OTUs	RA%
***Pseudomonadota***	605	19.4	266	22.8	370	16.2	337	19.8
***Bacillota***	519	16.6	222	19	393	17.2	330	19.4
***Bacteroidota***	447	14.3	179	15.4	305	13.4	254	14.9
***Planctomycetota***	203	6.5	51	4.4	162	7.1	84	4.9
***Patescibacteria***	119	3.8	37	3.2	90	3.9	48	2.8
***Spirochaetota***	116	3.7	38	3.3	96	4.2	62	3.6
***Actinomycetota***	113	3.6	61	5.2	67	2.9	66	3.9
***Chloroflexota***	104	3.3	41	3.5	84	3.7	62	3.6
***Kiritimatiellaeota***	98	3.1	25	2.1	84	3.7	59	3.5
***Mycoplasmatota***	87	2.8	31	2.7	60	2.6	46	2.7
**Unidentified**	81	2.6	14	1.2	68	3	22	1.3
**Others**	628	20.1	201	17.2	502	22	330	19.4
Grand Total	3,120	100	1,166	100	2,281	100	1700	100

Others: Verrucomicrobiota, Halanaerobiaeota, Gemmatimonadota, BHI80-139, Omnitrophicaeota, Cloacimonetes, Acidobacteria, Elusimicrobiota, Hydrogenedentes, Chlamydiota, Deinococcota, Lentisphaerota, uncultured, BRC1, Cyanobacteria, WS1, Dependentiae, Fibrobacterota, Thermotogota, Armatimonadota, Fusobacteriota, Acetothermia, Synergistota, Margulisbacteria, Atribacterotaia, Epsilonbacteraeota, LCP-89, TA06, Caldisericota, CK-2C2-2, Latescibacteria, MAT-CR-M4-B07, Modulibacteria, WOR-1, Chrysiogenota, FCPU426, PAUC34f, Rokubacteria, GN01, WS2. *RA: Relative abundance as proportion out of the total.

In addition, *Cyanobacteria* were one of the most prevalent phyla in the three lakes studied (Figure 4; Supplementary Table 2). *Cyanobiaceae* (*Cyanobium*), *Phormidiaceae* (*Arthrospira*), *Cyanobacteriaceae* (*Cyanobacterium*), *Nostocaceae* (*Nodularia*), and *Paraspirulinaceae* (*Spirulina*) were the prevalent taxa in the lakes within the phylum *cyanobacteria* (Supplementary Table 2).

*Halomonadaceae*, *Ectothiorhodospiraceae*, and *Idiomarinaceae* were the most abundant families of phylum *Pseudomonadota*, with *Halomonadaceae* as the most abundant family in lakes Abijata and Shala. At the same time, *Syntrophomonadaceae* were the most abundant family in lake Chitu. Furthermore, *Bacillota* (*Bacillaceae*), *Rhodothermia* (*Bacteroidota*), and *Nitriliruptoria* (*Actinomycetota*) were among the most abundant taxa (Supplementary Table 3).

About 37.4% of OTUs were unidentified in the Silva database at the species level. Most of the identified species are listed as uncultivable bacterial species (Supplementary Table 2).

### Archaeal community composition

In samples from Lake Chitu, seven archaeal phyla were present, with two phyla, *Euryarchaeota* and *Nanoarchaeaeota,* accounting for 88% of the OTUs (Table 3). In samples from Lake Abijata, five archaeal phyla were detected. Again, *Euryarchaeota* and *Nanoarchaeaeota* were dominant, like in Lake Chitu, accounting for 91% of the OTUs. In samples of Lake Shala, the phylum *Nanoarchaeaeota* was the only archaea detected (Table 3).

**Table 3:** Archeael phyla distribution among the lakes identified from environmental samples through culture-independent investigation.

**Phylum**	**Number Of OTUs**							
	All three lakes		Abijata		Chitu		Shala	
	No.	RA (%)	No.	RA (%)	No.	RA (%)	No.	RA (%)
***Altiarchaeota***	4	0.8	0	0	4	1	0	0
***Asgardaeota***	9	1.9	0	0	9	2.2	0	0
***Crenarchaeota***	24	5	6	5.5	20	4.9	0	0
***Diapherotrites***	8	1.7	2	1.8	8	2	0	0
***Euryarchaeota***	114	23.7	53	48.2	74	18.2	0	0
***Hadesarchaeaeota***	9	1.9	2	1.8	7	1.7	0	0
***Nanoarchaeaeota***	313	64.9	47	42.7	283	69.7	9	100
**Unidentified**	1	0.2	0	0	1	0.3	0	0
Grand Total	**482**	**100**	**110**	**100**	**406**	**100**	**9**	**100**

*RA: Relative abundance (Proportion out of the total).

The *Thermoplasmata*, *Thermococci*, *Methanomicrobia*, and *Halobacteria* were the most abundant groups of *Euryarchaeota*. More than 96% of the *Nanoarcheota* belonged to the class *Woesearchaeia*, and the remaining 4% was unclassified. In samples of Lake Abijata, 60% of *Euryarchaeota* belonged to *Halobacteria,* whereas in samples of Lake Chitu, 78% of OTUs of *Euryarchaeota* belonged to *Thermoplasmata* and *Methanomicrobia* (Supplementary Table 3). Around 39% of the archaeal sequences were unidentified archaeal species, and more than 60% of the sequences were listed as uncultured (Supplementary Table 2).

### Eukaryotic community composition

By ITS sequencing, a total of 1,0250,22 eukaryotic reads were identified. These reads were categorized into 898 OTUs (Table 4). About 51% of OTUs were represented in samples of Lake Abijata, followed by 37% in samples of Lake Shala. No eukaryotic sequences were detected in Lake Chitu sediment samples (Table 4). The alpha diversity showed that the Lake Shala sediment sample (SHALAK) had the highest species diversity (Figure 2C). All eukaryotic reads were identified to be fungal.

**Table 4:** The obtained eukaryotic OTUs and sequence of the environmental samples identified through culture-independent investigation.

Lake	OTUs detected		Number of reads		Total OTUs	Total Reads	% Reads	Unique OTUs*	Shared OTUs	% OUT Occurrence***
	Water	Sediment	Water	Sediment				(%)	(%)	
Abijata	199	320	467,937	521,659	460	9,89,596	71.6	37.9	13.4	51.2
Chitu	293	0	152,889	0	293	1,52,889	11	21.3	11.4	32.6
Shala	87	271	94,098	146,021	334	2,40,119	17.4	25	12.5	37.2
Total	898		1,025,022		898	1,025,022	100	84.1	15.9	100

*Unique OTUs refer to those that are detected only in one lake.

**Shared OTUs refer to those that occur in two or all three lakes.

***OTUs occurrence refers to the proportion of OTUs detected in each lake out of the total OUT detected.

### Shared and unique fungal OTUs within and between samples of the lakes

**Figure 5:**
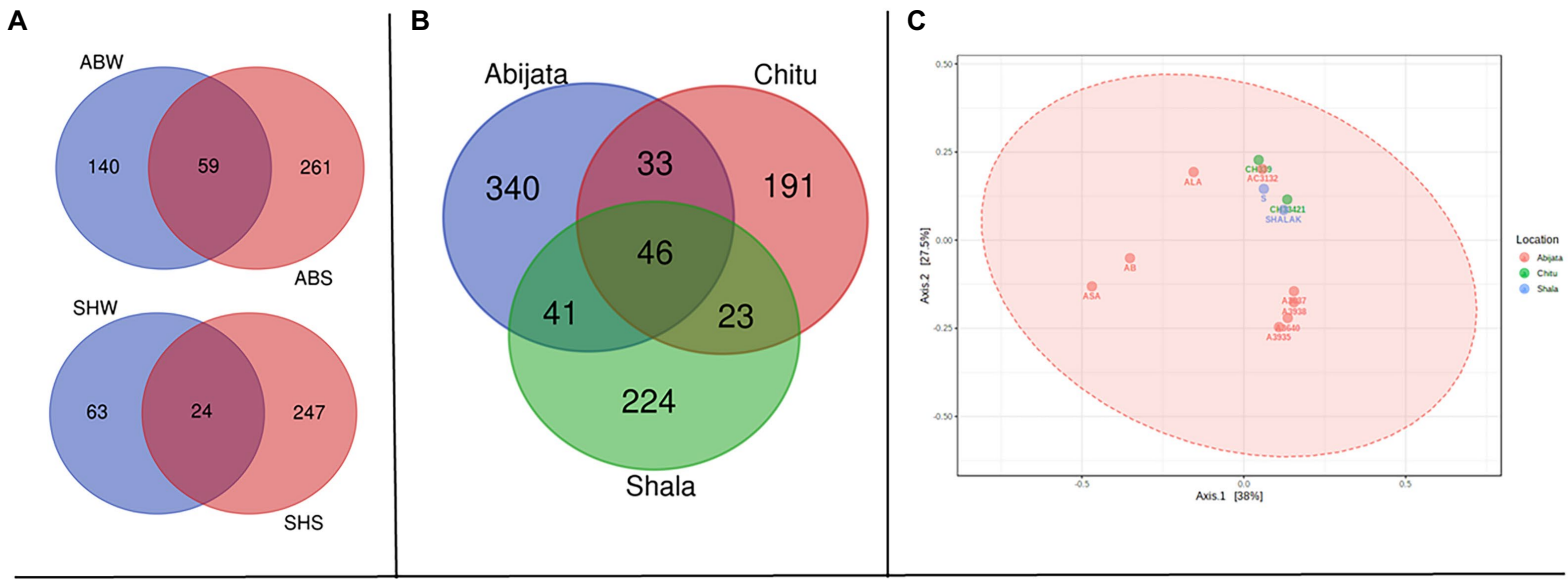
Shared OTUs between **(A)** sample types; ABW: Abijata water samples, ABS: Abijata Sediment samples, SHW: Shala water samples, SHS: Shala sediment samples **(B)** between the lakes **(C)** PCoA plot using Bray distance. The explained variances are shown in brackets (F-value: 1.1663; R-squared: 0.20583; *p* > 0.05; Sediment Samples: A3640, A3935, A3938, AB, SHALAK; Water Samples: A3637, AC3132, ALA, ASA, CH33421, CH339, S).

Sediment samples of Lake Abijata and Lake Shala had a higher number of unique fungal OTUs than water samples from the same lakes (Figure 5A). Only 46 OTUs were shared between samples from all three lakes, with samples of Lake Chitu and Lake Shala sharing 23 OTUs (Figure 5B). One-way ANOVA analysis showed a significant difference between OTUs of the lakes (*F*-value = 9.0, value of *p* <0.05). The PCoA showed a similar variation of samples from Lake Shala and Lake Chitu, with positive values on both principal coordinates, while some of the Lake Abijata samples clustered with positive first principal coordinate values and negative second coordinate values (Figure 5C).

### Fungal community taxonomic distribution

The ITS sequence reads of the fungal communities were categorized into four phyla, where *Ascomycota* and *Basidiomycota* were the most abundant in all lake samples. All the identified eukaryotic phyla were found in samples of Lakes Abijata and Shala. Lake Shala had the highest proportion of unidentified sequence reads (>17%; Table 5).

**Table 5:** Fungal phylum composition of environmental samples identified through culture-independent investigation.

**Phylum**	**All of the lakes**		**Abijata**		**Chitu**		**Shala**	
	OTUs	RA (%)	OTUs	RA (%)	OTUs	RA (%)	OTUs	RA (%)
***Ascomycota***	454	50.56	225	48.9	161	55	180	53.9
***Basidiomycota***	283	31.51	154	33.5	102	34.8	92	27.5
***Chytridiomycota***	7	0.78	6	1.3	0	0	1	0.3
***Rozellomycota***	3	0.33	1	0.2	0	0	2	0.6
**Unidentified**	151	16.82	74	16.1	30	10.2	59	17.7
Total	898	100	460	100	293	100	334	100

All the fungal communities were categorized into 19 classes. The *Dothideomycetes*, *Saccharomycetes,* and *Sordariomycetes* from phylum *Ascomycota*; *Agaricomycetes*, *Tremellomycetes,* and *Malasseziomycetes* from phylum *Basidiomycota* were the abundant groups, respectively. More than 50% of fungal OTUs were not identified at the species level (Supplementary Table 4).

### Enrichment cultures

Enrichment cultures yielded a total of 134 OTUs, which belonged to five phyla. Of these, *Pseudomonadota*, *Bacillota* and *Bacteroidota* accounted for more than 95% of the identified OTUs. No archaeal or eukaryotic groups were identified in the enrichment samples (Table 6). *Pseudomonadota* were represented by a higher number of OTUs in Lake Chitu and Shala samples, whereas Bacillota was the most abundant phylum in Lake Abijata (Table 6).

**Table 6:** The Phylum distributions identified through enrichment culture study.

Phyla	All		Abijata		Chitu		Shala	
	OTUs	OTUs (%)	OTUs	OTUs (%)	OTUs	OTUs (%)	OTUs	OTUs (%)
Actinomycetota	1	0.75	1	1.1	1	1.7	1	1.6
Bacteroidota	33	24.6	23	24.5	8	13.8	16	25.0
BacillotaBacillota	47	35.1	34	36.2	16	27.6	23	35.9
Pseudomonadota	48	35.8	32	34.0	32	55.2	24	37.5
Mycoplasmatota	5	3.7	4	4.3	1	1.7		0.00
Grand Total	134	100	94	100	58	100	64	100

In total, only 3.0% of the OTUs identified in the culture-independent (environmental) samples, were detected in the enrichment samples, all of which were bacterial OTUs. Thus, out of the 50 bacterial phyla that were identified from environmental samples, only 5 phyla were detected in the enrichment cultures (Table 7). Three phyla, *Pseudomonadota*, *Bacillota*, and *Bacteriodetes* with high abundance in the environmental samples were also the most abundant in the enrichment cultures (Table 7).

**Table 7:** Comparison of OTUs detected in through culture-independent and enrichement culture studies.

	Total OTU detected		% grown in culture
**Phylum**	**Environmental samples**	**Enrichement samples**	
**Bacteria**			
Pseudomonadota	605	48	7.9
Bacillota	519	47	9.1
Bacteroidota	447	33	7.4
Planctomycetota	203	0	0
Patescibacteria	119	0	0
Spirochaetota	116	0	0
Actinomycetota	113	1	0.9
Chloroflexota	104	0	0
Kiritimatiellaeota	98	0	0
Mycoplasmatota	87	5	5.7
Unidentified	81	0	0
Others	628	0	0
**Total Bacteria**	**3,120**	**134**	**4.3**
**Archaea**			
Altiarchaeota	4	0	0
Asgardaeota	9	0	0
Crenarchaeota	24	0	0
Diapherotrites	8	0	0
Euryarchaeota	114	0	0
Hadesarchaeaeota	9	0	0
Nanoarchaeaeota	313	0	0
Unidentified	1	0	0
**Total Archaea**	**482**	**0**	**0**
**Unassigned prokaryote 1 0 0**			
**Eukaryotes**			
Ascomycota	454	0	0
Basidiomycota	283	0	0
Chytridiomycota	7	0	0
Rozellomycota	3	0	0
Unidentified	151	0	0
**Total Eukaryotes**	**898**	**0**	**0**
**TOTAL**	**4,501**	**134**	**3**

Further analysis of taxa levels found that more than 83% of the *Pseudomonadota* were *Gammaproteobacteria*, in which *Idiomarinaceae* and *Halomonadaceae* accounted for 60%. In the *Bacillota* phylum, more than 80% of the OTUs belonged to the *Bacilli* class, where the remaining was *Clostridia.* Furthermore, at the species level 46.5 and 16.2%, belonged to unidentified and ambiguous taxa, respectively (Supplementary Table 5).

## Discussion

We observed high prokaryotic and eukaryotic microbial diversity from the three Ethiopian soda lakes using amplicon sequencing. The current study also demonstrated the challenges of capturing these unique microbes, which are adapted to extreme environments, using typical enrichment cultures. Although previous studies have also reported high biodiversity of microorganisms in the Ethiopian soda lakes (Lanzén et al., 2013; Simachew et al., 2016), significantly higher levels were found in the present study. We identified a total of 3,603 prokaryotic and 898 eukaryotic OTUs in the samples from the three soda lakes, which is a significantly higher number than the 2,704 OTUs reported in a previous study from five Ethiopian soda lakes, including the three lakes studied here (Lanzén et al., 2013). In fact, the OTU richness observed in this study was also higher than that reported previously for other hypersaline lakes (Dimitriu et al., 2008b; Zorz et al., 2019). Here, we used high-abundance short-read sequencing for the estimation of the microbial diversity to circumvent the shortcomings of sequencing methods with low coverage and sequence depth used in previous studies of the Ethiopian soda lakes. However, the three investigated soda lakes differed in microbial biodiversity; 75% of all OTUs detected in the three lakes were present in samples of Lake Chitu, while only 35 and 14% of the OTUs were present in samples of Lake Abijata and Lake Shala, respectively. This can be explained by the differences in the primary production of the lakes (Ogato et al., 2015). The East African Rift Valley soda lakes are among the world’s most productive aquatic systems with primary production rates exceeding 10 g C m^−2^ day^−1^ (Grant and Jones, 2016), providing abundant organic matter supporting a diverse group of microorganisms. Lake Chitu is the most productive among the three lakes, having thick biomass of the blue-green algae, *Arthrospira fusiformis* (Faris, 2017).

In the present study, samples of Lake Chitu and Lake Abijata shared the highest number of prokaryotic OTUs, while in previous studies, a higher overlap of OTUs was observed between Lake Shala and Lake Abijata (Lanzén et al., 2013). This discrepancy in overlaps might result from the fourfold salinity increment in Lake Abijata, which is a higher increase than in the other Ethiopian soda lakes over the last three decades (Ayenew and Legesse, 2007; Klemperer and Cash, 2007; Faris, 2017). During the last three decades, the surface area of Lake Abijata has decreased by more than 50% and the average depth by 5 m as a result of the abstraction of water for soda ash production by the Abijata-Shala Soda Ash Factory and the diversion of the lake’s feeder rivers, Horakelo and Bulbula, for irrigation (Ayenew and Legesse, 2007; Simachew et al., 2016). Several studies have shown that a change in salinity is an important environmental factor affecting the abundance and diversity of microorganisms, eliminating some and benefiting other groups (Lozupone et al., 2007; Xiong et al., 2012). A change in the microbial composition of Lake Abijata over time has been reported in previous studies (Simachew et al., 2016). Thus, salinity might be a critical factor in structuring the prokaryotic communities of haloalkaline environments, as also indicated by the present study.

Bacteria accounted for the vast majority of the prokaryotic OTUs identified in this study (87%), with Archaea accounting for only a small fraction (13%). The primary explanation for this might be that these lakes are moderately saline soda lakes with brine salinities ranging from 50 to 250 g/l (Sorokin et al., 2014). Previous research on microbial diversity in moderately saline soda lakes found that these ecological niches have more diverse archaea and bacteria communities than less and high saline environments, with bacteria dominating (Felföldi, 2020).

In line with previous studies from low and moderate saline soda lakes, *Gammaproteobacteria* (including the genus *Halomonas*), *Bacillota* (*Bacillus, Clostridia*), *Bacteroidota* (*Cytophaga*, *Flavobacterium*, *Bacteroides*), and *Rhodobacteraceae* (Humayoun et al., 2003; Dimitriu et al., 2008a; Asao et al., 2011; Lanzén et al., 2013; Sorokin et al., 2014) were the prevailing prokaryotes in this study as well. The groups *Halomonas*, *Bacillota*, and other heterotrophic bacteria are primarily responsible for immediate degradation of organic matter produced by autotrophic bacteria like Cyanobacteria (Jones and Grant, 1999; Sorokin et al., 2014). *Halomonas* have been reported to have biotechnological potential in the production of exopolysaccharides, enzymes, and compatible solutes such as ectoine (Ye and Chen, 2021). In addition, *Halomonas* has an active role in denitrification and the degradation of aromatic compounds (Tahrioui et al., 2013). *Rhodobacteraceae*, another abundant microbial taxon in the soda lakes, comprises aerobic photo- and chemoheterotrophs and purple non-sulfur bacteria, which perform photosynthesis in anaerobic environments. They are deeply involved in sulfur and carbon biogeochemical cycling and are in symbiosis with aquatic micro- and macroorganisms (Pujalte et al., 2014).

Environmental heterogeneity with more ecological niches has been pointed out as an important variable for high microbial biodiversity (Wobus et al., 2003). Such heterogeneity has been reported as the reason for higher biodiversity in sediment than in corresponding water samples (Zinger et al., 2011; Banda et al., 2020), which was also the case in the present study. In this study, the sediment samples from Lake Abijata and Lake Shala showed higher levels of prokaryote and eukaryote OTUs than the corresponding water samples. Furthermore, sediment samples from Lake Abijata and Lake Shala were taken as lake surface sediment samples. Lake surface sediments contain abundant and diverse microbial populations (Yang et al., 2016), and microorganisms in lake surface sediments play vital roles in regulating nutrient dynamics and biogeochemical cycles (Zhang et al., 2020). In contrast to Lake Abijata and Lake Shala, and to previous results (Tafesse, 2014), Lake Chitu showed a higher number of prokaryotic OTUs in the water samples than the sediment samples. This might be attributed to abundant algal biomass and other organic components in the water of Lake Chitu that sustain diverse microbial communities (Ogato et al., 2015). In samples of Lake Abijata, *Halobacteria* was the most abundant class, possibly because this lake had higher salinity compared to the other two lakes during our sampling. Hypersaline lakes have in previous studies, been reported to be dominated by halophilic archaea belonging to the class *Halobacteria* (Ochsenreiter et al., 2002; Mesbah et al., 2007).

The archaeal OTUs in the samples from Lake Shala and Chitu were associated to the *Nanoarchaeaeota* phylum that belongs to the class of *Woesearchaeia*. These are likely anaerobic, fermentative, and syntrophic archaea and are often found in marine environments with high organic matter content (Gründger et al., 2019). However, it is unknown how geochemical circumstances shape the distribution pattern of *Woesearchaeia* and their ecological role, particularly on a global scale (Xiao et al., 2020). *Woesearchaeia* can enable carbon and hydrogen metabolism in anoxic circumstances, but they are also associated with symbiotic or parasitic lifestyles, which is reflected in their small genome sizes and limited metabolic capabilities. Furthermore, certain reports imply that this archaeal group plays an essential role in iron and methane biogeochemical cycles (Liu et al., 2018; Gründger et al., 2019).

*Thermoplasmata*, *Methanomicrobia*, and *Thermococci* were dominant microbial prokaryotes in phylum *Euryarchaeota* of these lakes. Previous studies have explored the methane cycle in soda lakes as an essential part of the microbial carbon cycle (Sorokin et al., 2014), and *Thermoplasmata* (Poulsen et al., 2013), *Thermococci* (Evans et al., 2019), *Methanomicrobia* (Lee et al., 2014), and *Halobacteria* (Sorokin et al., 2017) have been evaluated for their role in methane cycling. The abundance of microorganisms involved in methane metabolism identified together with the presence of cyanobacteria points at high concentrations of dissolved biogenic methane in these lakes. In anoxic lake sediments, the abundant photosynthetic biomass eventually undergoes methanogenic degradation (Fazi et al., 2021).

While archaea and bacteria are known for their ability to adapt to extreme environments, fungi are generally not expected in saline soda lakes, since they generally prefer acidic or neutral pH (Grum-Grzhimaylo et al., 2016). However, recent studies have shown that eukaryotic microorganisms, including fungi, can endure or even thrive in extreme environments. The fungal diversity of the Ethiopian soda lakes has been rarely studied previously except from the report of Lanzén et al., 2013. In the present study, fungi were found in samples of all three lakes, with *Ascomycota* as the most abundant phylum in all three lakes. The Ascomycota orders *Capnodiales*, *Dothideales*, *Cladosporium*, *Alternaria*, and *Eurotiales* have previously been found in different extreme environments globally, including saline lakes, soda lakes, and in Arctic glacier ice (Cantrell et al., 2006; Anne et al., 2016; Fotedar et al., 2018; Orwa et al., 2020). Specifically, the detection of *Malassezia* in the soda lake samples studied here extends previous culture-independent studies of fungi that have shown that *Malassezia* is exceedingly widespread and ecologically diverse (Boekhout et al., 2010). Recent studies in little-characterized marine environments point to extensive diversification of Malassezia-like organisms, suggesting further opportunities to explore this group’s ecology, evolution, and diversity (Amend, 2014).

The present study showed that culturing captures only a small proportion of naturally occurring microbial biodiversity; out of a total of 4,500 OTUs detected by sequencing of environmental samples, only 134 OTUs (representing 3% of the total) grew in enrichment cultures. Previous studies have indicated that since most microorganisms are impossible or difficult to culture, standard culture-dependent techniques provide information on 1% or less of the microbial diversity in a given environmental sample (Coughlan et al., 2015). One reason for this is that culturing cannot reproduce the natural condition of the ecological niches where samples are collected (Carraro et al., 2011). For example, the media selected for microbial cultivation have been shown to selectively favour a small fraction of the microbial diversity (Carraro et al., 2011; Al-Awadhi et al., 2013). To minimize the selectivity of the culture media due to, for example, lack of critical trace elements, we used lake water from the three lakes to mimic the natural environment in our enrichment culture study. Nevertheless, even under such conditions, 97% of the OTUs detected in the environmental samples could not be detected after cultivation. Since we used a mixed liquid culture, quorum sensing is probably not responsible for the failure of most organisms to grow (Bodor et al., 2020). In their natural habitat, these microorganisms are exposed to different concentrations of nutrients and oxygen due to, for example, the depth of the water and diurnal fluctuations, which are impossible to reproduce in the laboratory. Thus, our results clearly showed the importance of culture-independent methods in studying microbial diversity.

High microbial diversity under polyextremophilic conditions (high salt and alkaline pH), as observed here, is important for possible industrial applications. Enzymes that are active and stable under alkaline conditions and in the presence of high salt concentration potential applicability in different industrial processes, as shown by previous microbial cultivation and screening studies that have resulted in the production of novel enzymes from Ethiopian soda lakes (Gessesse and Gashe, 1997; Martins et al., 2001; Haile and Gessesse, 2012). Targeted sequencing of enzyme groups or whole genome sequencing will be powerful tools to secure unique enzymes with specific properties.

## Conclusion

This study showed that the amplicon sequencing utilized was highly effective in detecting a high degree of microbial diversity in three samples from East African Rift Valley soda lakes, suggesting that increased sampling covering all microhabitats of these soda lakes would detect considerably more organisms. This study also showed the presence of eukaryotic microorganisms, such as fungi, in soda lakes. In addition, despite our attempts to replicate the natural environment for microbe cultivation, only a small fraction of microorganisms detected by sequencing could be cultured in the laboratory. Additional culture-independent studies are needed to successfully exploit the full potential of microorganisms, including the unculturable microbes, in soda lakes.

## Data availability statement

The data presented in the study are deposited in the NCBI repository with accessions PRJNA816843 and PRJNA817405. This data can be found at: https://www.ncbi.nlm.nih.gov/sra/PRJNA816843 and https://www.ncbi.nlm.nih.gov/sra/PRJNA817405.

## Author contributions

All authors contributed to the study conception and design. Experiment was designed by OJ, AG, AS, EJ, and EA. Data collection and analysis were performed by OJ. The first draft of the manuscript was written by OJ. All authors commented on previous versions of the manuscript. All authors contributed to the article and approved the submitted version.

## Funding

This study was financed by the Swedish International Development Cooperation Agency (SIDA) through the research and training grant awarded to Addis Ababa University and the Swedish University of Agricultural Sciences (AAU-SLU Biotech)^2^ and FORMAS (2019–00527).

## Conflict of interest

The authors declare that the research was conducted in the absence of any commercial or financial relationships that could be construed as a potential conflict of interest.

## Publisher’s note

All claims expressed in this article are solely those of the authors and do not necessarily represent those of their affiliated organizations, or those of the publisher, the editors and the reviewers. Any product that may be evaluated in this article, or claim that may be made by its manufacturer, is not guaranteed or endorsed by the publisher.

## Footnotes

^1^ https://github.com/nf-core/ampliseq

^2^ https://sida.aau.edu.et/index.php/biotechnology-phdprogram
